# Comparing multiband and singleband EPI in NODDI at 3 T: what are the implications for reproducibility and study sample sizes?

**DOI:** 10.1007/s10334-020-00897-7

**Published:** 2020-12-14

**Authors:** Samira Bouyagoub, Nicholas G. Dowell, Matt Gabel, Mara Cercignani

**Affiliations:** 1 Clinical Imaging Sciences Centre, Department of Neuroscience, Brighton and Sussex Medical School, University of Sussex, Falmer, Brighton, BN1 9RR UK; 2 Neuroimaging Laboratory, Santa Lucia Foundation, Via Ardeatina 306, 00179 Rome, Italy

**Keywords:** NODDI, Diffusion MRI, Multiband EPI, Reproducibility

## Abstract

**Objective:**

The reproducibility of Neurite orientation dispersion and density imaging (NODDI) metrics from time-saving multiband (MB) EPI compared with singleband (SB) has not been considered. This study aims to evaluate the reproducibility of NODDI parameters from SB and MB acquisitions, determine the agreement between acquisitions and estimate the sample sizes required to detect between-group change.

**Methods:**

Brain diffusion MRI data were acquired using SB and MB (acceleration factors 2 (MB2) and 3 (MB3)) on 8 healthy subjects on 2 separate visits. NODDI maps of isotropic volume fraction (FISO), neurite density (NDI) and orientation dispersion index (ODI) were estimated. Region-of-interest analysis was performed; variability across subjects and visits was measured using coefficients of variation (CoV). Intraclass correlation coefficient and Bland–Altman analysis were performed to assess reproducibility and detect any systematic bias between SB, MB2 and MB3. Power calculations were used to determine sample sizes required to detect group differences.

**Results:**

Both NDI and ODI were reproducible between visits; however, FISO was variable. All parameters were not reproducible across methods; a systematic bias was observed with the derived values decreasing as the MB factor increases. The number of subjects needed to detect a between-group change is not significantly different between methods; however, ODI needs considerably higher sample sizes than NDI.

**Conclusions:**

Both SB and MB yield highly reproducible NDI and ODI measures, but direct comparison of these parameters between methods is complicated by systematic differences that exist between the two approaches.

**Supplementary Information:**

The online version contains supplementary material available at 10.1007/s10334-020-00897-7.

## Introduction

Diffusion MRI (dMRI) is an established non-invasive MRI technique that is instrumental in characterising tissue microstructure by probing the diffusion properties of water molecules within the tissue over distances of a length scale comparable to that of cellular structures [1]. Several advanced diffusion models have been developed over the years to generate indices that quantify specific tissue properties relating to the geometry and organisation of neurites [2]. In contrast to the standard diffusion tensor imaging (DTI, [3]), these complex models require larger datasets acquired at multiple *b* values and higher angular resolution, often resulting in prohibitively long acquisition times for clinical applications. Among these models, neurite orientation dispersion and density imaging (NODDI) [4] has become very popular amongst multicompartment dMRI models, as it was designed to characterise axons and dendrites, within clinically feasible acquisition times. NODDI combines a hierarchical three-compartment model with a high angular resolution diffusion-weighted imaging (HARDI) protocol to differentiate the MRI signal from tissue and free water (isotropic compartment) within a voxel and intraneurite and extraneurite space within the tissue compartment.

NODDI estimates a number of quantitative parameters that characterise tissue microstructure voxelwise across the image. Orientation dispersion index (ODI) describes the organisation and orientation of neurites (axons and dendrites), neurite density index (NDI) is derived from the intraneurite volume fraction within the tissue compartment of a voxel and the volume fraction that undergoes isotropic diffusion (FISO) which is generally assumed to represent the CSF compartment within a voxel. NODDI also estimates fibre orientation vectors. NODDI’s ability to decouple NDI and ODI helped shed more light on the source of diffusion anisotropy, previously quantified in conventional DTI using the fractional anisotropy index (FA) [5]. This is particularly useful in the regions of crossing fibres which are often problematic for DTI [6]. However, the NODDI model has also attracted some criticism regarding the use of some assumptions that, if violated, can result in a bias in its parameters [7, 8]. These include the assumption of a common T2 for all compartments [9] and a common default value for the intrinsic diffusivity that is reasonable for white matter tissue but sub-optimal in gray matter [10, 11]. Although the model has some limitations, it is important to acknowledge its advantages in terms of feasibility, and the recent analysis shows agreement between NODDI metrics and histologically equivalent metrics [12]. NODDI has found a number of applications in neuroimaging from studying multiple sclerosis [12, 13], Alzheimer’s disease [14] and healthy neurodevelopment [15], to characterising myelination [16, 17], inflammation [18] and first-episode psychosis [19]. As NODDI usage increases, efforts have been made to establish the reliability and reproducibility of its indices. It has been demonstrated that NODDI is sensitive to field strength [20] and acquisition parameters such as the maximum *b* value and the number of diffusion-encoding directions [21], although the impact varies depending on the anatomical region of interest.

NODDI data can be acquired in clinically feasible times using the conventional echo planar imaging (EPI) approach that involves exciting a single slice at a time (singleband SB). However, with the introduction of the multiband (MB) or simultaneous multislice EPI [22, 23], the acquisition time for NODDI can be reduced further. In the MB technique, acquisition is accelerated by simultaneously exciting multiple slices using a single radio-frequency (RF) pulse, without significantly compromising the spatial resolution or the signal-to-noise ratio (SNR). By increasing the number of simultaneously excited slices, known as the MB factor, it is possible to reduce the repetition time (TR) and hence the total acquisition time. However, a known issue with multiband is the non-uniform noise caused by the geometrical arrangement of the receiver coils (the *g* factor) and this causes SNR to be different across different brain regions [24]. Although MB was introduced to increase temporal resolution for functional MRI [25], diffusion MRI can also benefit by significantly reducing the scan time. MB has already been incorporated in standard dMRI protocols such as the Human Connectome Project's diffusion MRI scanning protocol, which uses MB with acceleration factor 3. It is known that MB comes at a price of reduced SNR and increased T1-weighting and with the increased usage of MB in combination with NODDI it is important to study the effects of MB sequences on diffusion data and derived diffusion metrics. Duan et al. [26] investigated the reliability of MB-derived DTI measures and showed moderate to good repeatability, which varied between ROIs depending on their size and location; but this was a test–retest study and did not compare the results with SB-derived measures. Another work by Mitsuda et al. [27] examined the effects of MB-EPI sequence (MB factors of 2,3 and 4) on DTI measures from data acquired using 1.5 T scanner and 12 channel head coil compared with SB-EPI data. This study showed significant differences in FA and ADC between SB data and MB data with higher acceleration factors of 3 and 4. Bernstein et al. [28] conducted a bootstrap analysis to compare diffusion metrics derived from SB with those derived from MB (factor 3) both at similar and reduced TR in order to study the effects of MB reconstruction and TR shortening separately. The study revealed a bias in the MB-derived maps and demonstrated an increase in uncertainty for each parameter when the TR is short. Olson et al. [29] examined the effects of slice crosstalk on diffusion parameters in simultaneous multislice imaging. They found that interslice leakage between simultaneously excited slices had an effect on the reproducibility of diffusion metrics from higher level dMRI models more than DTI metrics.

These findings, combined with the observation that MB potentially introduces artefact, [30] and a signal-to-noise penalty, suggest that maps derived from NODDI might also be affected.

In this paper, we investigate the reproducibility of the NODDI indices and explore the intersession and intersubject variations in healthy volunteers, determine the agreement between MB and SB acquisition methods and to establish any systematic differences between MB and SB derived parameters. Finally, we perform power calculations in order to estimate the sample sizes required to detect a between-group change for both acquisition methods.

## Materials and methods

### Subjects and scan sessions

Eight healthy participants were recruited in this study: 7 male, median age 34 (range 22–40) years. Each participant was scanned on two separate visits, arranged between 2 and 7 days apart. We limited the gap between visits to a minimum of 2 days and maximum of 7 days; this is short enough to ensure that there is no physiological change in the participants for DWI, but long enough to take into account the different state of the scanner that is a potential source of variability. Each visit included NODDI acquisition with MB2 (Multiband with acceleration factor 2), MB3 (Multiband with acceleration factor 3) and conventional single band (SB). This study falls within the ethical approval granted as part of a larger methodological development study approved by the Brighton and Sussex Medical School Ethics Committee; all participants provided written informed consent.

The images were acquired using a Siemens 3 T Prisma scanner (Siemens, Erlangen, Germany) with a maximum gradient strength of 80 mT/m and a 32-channel head coil. The same pulse sequence developed by the University of Minnesota Center for Magnetic Resonance Research was used to acquire single band (SB) and MB data (sequence version R016a). Diffusion-weighted data were acquired with single-shot, twice-refocused pulsed gradient spin-echo echo EPI using acquisition parameters that are typically used in NODDI studies. Sequence parameters were: TR = 7210, 4100 and 2800 ms for SB, MB2 and MB3 acquisitions, respectively; echo time (TE) = 82.80 ms; field of view = 240 × 240 (mm2); matrix size = 96 × 96; number of slices = 60; slice thickness = 2.5 mm; total acquisition time = 13, 9 and 7 min for SB, MB2 and MB3 data, respectively. Two *b* value shells were acquired with *b* = 800 and 2600 s/mm2, with 30 and 60 non-colinear diffusion-weighted directions, respectively. Eight volumes with no diffusion weighting (i.e. *b*=0) were acquired (b0 images). Further b0 images were acquired in the opposite phase encoding direction in order to estimate and correct for susceptibility induced distortions [31]. Images were acquired using generalised auto-calibrating partially parallel acquisition (GRAPPA, reduction factor=2), which not only reduces scan time, but also improves image quality by reducing EPI signal distortions.

### Image analysis

**Fig. 1:**
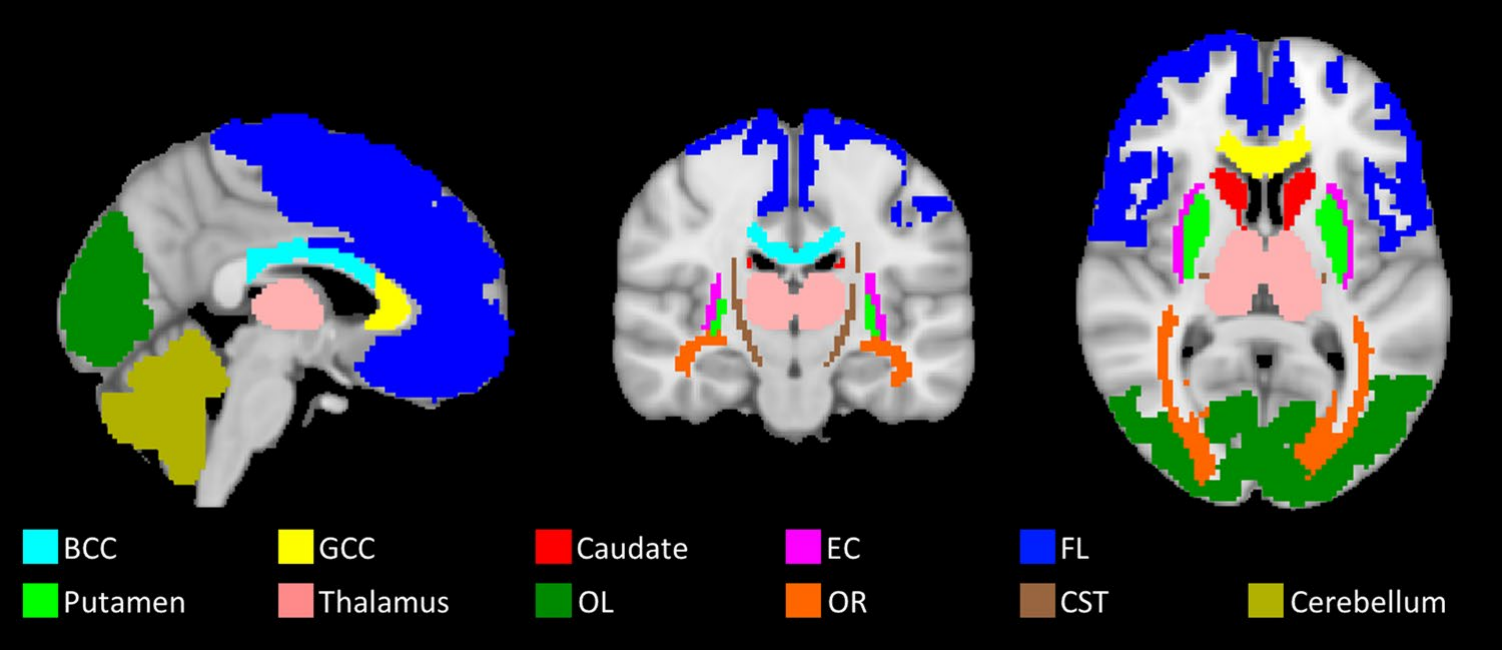
The regions selected for region of interest analysis for this study: *BCC* body of corpus callosum, *GCC* genu of corpus callosum, *CST* corticospinal tracts, *EC* external capsules, *OR* optic radiation, *FL* frontal lobe, *OL* occipital lobe, caudate, putamen thalamus and cerebellum

All diffusion-weighted images were first corrected for movement and eddy current distortions using FMRIB software library (FSL, version 5.0.7, Oxford, UK). FSL’s topup tool was used to correct for susceptibility and FSL’s Eddy command was used to correct for eddy current distortions [32]. The corrected data were then fitted to the NODDI model using the toolbox (http://mig.cs.ucl.ac.uk/mig/mig/index.php/?n=Tutorial.NODDImatlab/) run in Matlab 2012b (The MathWorks, Inc., Natick, MA) using a high performance computing cluster of 128 cores to generate voxelwise whole brain maps of FISO, NDI and ODI. The resulting NODDI parameter maps were normalised to the Montreal Neurological Institute (MNI) space using the Advanced Normalisation Tools (ANTs, version 2.1.0; http://stnava.github.io/ANTs) in order to perform region-of-interest (ROI) analysis. This involved calculating the diffeomorphic transformation required to warp the mean b0 image to the MNI152 T2-weighted image template, (with spatial resolution of 2x2x2 mm3). A selection of ROIs was chosen for analysis for which the mean and standard deviation was calculated for each NODDI parameter; the ROIs were obtained from the ICBM-DTI-81 white-matter labels atlas [33] for white matter regions and the non-linear MNI-ICBM152 atlas [34] for the gray matter regions. A selection of brain regions was chosen to reflect areas of different microstructural properties (e.g. fibre density) and challenges (e.g. partial volume effect). The ROIs selected for this study included body of corpus callosum (BCC), genu of corpus callosum (GCC), corticospinal tracts (CST), external capsules (EC) and optic radiation (OR) from the white matter. Although the frontal lobe (FL) and the occipital lobe (OL) were chosen to represent the cortical gray matter; and the caudate, putamen and thalamus were selected from the deep gray matter regions. The cerebellum was also included in this study. Figure 1 illustrates the size and location of these ROIs on the brain.

In order to perform the ROI-based statistical analyses, the mean values for the NODDI parameters NDI, FISO and ODI were extracted for each ROI.

### Comparing raw diffusion signal between SB and MB acquisition methods

In this section, we evaluate the impact of MB sequences on the raw diffusion signal (in the non-diffusion weighted and diffusion weighted images) prior to NODDI fitting and explore if any differences can be observed between MB-acquired and SB-acquired images. Before any quantitative comparison, a visual inspection was performed on the b0 images after movement and eddy distortion corrections and co-registration to identify the presence of image artefacts from either GRAPPA or MB acceleration techniques.

### SNR

Before performing any ROI-based image analysis on the NODDI metrics, a voxel-wise SNR calculation was performed for each NODDI dataset, using the MRtrix3 software [35]. This involves calculating the ratio of the mean signal and the standard deviation (SD) of the eight *b*=0 volumes. The resulting SNR map was used to calculate the mean SNR within each ROI. We tested for the statistical significance of the differences between mean SNR for SB, MB2 and MB3 using paired *t* test. The SNR maps were also visually inspected to identify any differences between SB, MB2 and MB3 approaches.

### Signal variability across diffusion weighting directions

Higher diffusion weighting is more sensitive to complex microstructure architecture resulting in greater signal variation. To account for that, NODDI requires the higher *b* value shell to be sampled at twice the angular resolution of the lower *b* value shell [4]. We investigated whether increasing the MB factor affects the variability between diffusion signal measured over different directions. To do this, we calculated the standard deviation for the signal at *b*=2600 across data acquired in all 60 directions and compared the results of SB with MB2 and MB3. In order to account for the intrinsic differences in signal intensity between SB, MB2 and MB3, the diffusion weighted images for each dataset were first normalised by dividing them by the dataset’s mean b0 image.

### Variability between subjects and between visits

To characterise the variability of the NODDI parameters, we used the coefficients of variation as a measure of variability between visits and subjects using the equation CoV (%) =100% × SD/mean.

Between-subjects variability: for each acquisition method (SB, MB2 and MB3), CoV between subjects within the data from the first visit was calculated.

Between-visits variability: for each acquisition method (SB, MB2 and MB3), we calculated the CoV between data from the first visit and the second visit. For this measure, SD is calculated between two measurements as: ${\text{SD}} = \sqrt {\frac{{\sum \left( {X1 - X2} \right)^{2} }}{2 \times N}}$ with: *N* being the number of the subjects and *X*1 and *X*2 being the two measurements (obtained from each ROI from visits 1 and 2, respectively) for each subject.

### Reproducibility between visits

The reproducibility of each NODDI measure obtained by each acquisition method between visits was quantified by means of the intraclass correlation coefficient (ICC) with the 95% confidence interval (CI). ICC estimates were calculated separately for SB, MB2, MB3, using NODDI parameters from visit 1 and visit 2. ICC considers both the within-subject variance due to measurement error and the variance because of biological differences between subjects. Ideally, the contribution from measurement error would be much smaller than from the subjects and in this case, ICC tends to 1. ICC estimates were calculated using SPSS statistical package version 25 (SPSS Inc, Chicago, IL), based on the single measures, absolute agreement, two-way mixed effects model. An ICC<0.50 was considered as poor reproducibility, 0.50–0.75 moderate, 0.75–0.9 good and >0.9 as excellent reproducibility, following the stratification introduced by Koo and Li [36].

### Agreement between acquisition methods

To determine the agreement between SB and MB acquisition methods, NODDI parameters resulting from these acquisitions are compared against each other using ICC as an index to reflect both correlation and agreement. ICC estimates were calculated across measurements within the same scanning visit (SB-versus-MB2, SB-versus-MB3 and MB2-versus-MB3); this was done using the NODDI measures from visit 1. Further to using ICC, Bland–Altman plots were also generated to compare SB with MB2, MB2 with MB3 and SB with MB3 using data from visit 1; a similar Bland–Altman analysis was also performed separately for data acquired in visit 2. The Bland–Altman plots show the difference between measurements against the mean of the measurements, the bias and the 95% limits of agreement (LoA). LoA for each NODDI parameter were defined as the mean of paired differences ± 1.95 × its standard deviation (SD). Bland–Altman plots make it possible to visualise the agreement between the acquisition methods, detect systematic bias and identify any relationship between the absolute differences and the mean value for each parameter [37]. Finally, in order to visualise and locate the origin of any possible bias in each metric, mean images across subjects for each NODDI metric from visit 1 were calculated for SB, MB2 and MB3 data and difference images were generated between SB- and MB-derived NODDI maps.

### Power and sample size calculations

Detecting physiological differences between groups (e.g. patients and controls) is a common aim for research studies that employ NODDI. Although the expected group differences can be estimated from previous (analogous) studies, it is not possible to determine the necessary group sizes to reliably detect these differences unless the reproducibility of the measurement technique is known. Here we use our quantification of reproducibility to determine the sample size required for detecting a reduction of 5% and 10% in NDI, ODI and FISO for each ROI using the mean and standard deviation (across subjects) of each NODDI measure from visit 1. The calculation was performed using Gpower [38], based on a *t* test involving the difference in the means between two independent groups with a two-tailed significance level of 0.05, power of 0.9 and equal sample sizes. Finally, we compared the estimated sample sizes for all parameters between methods (SB, MB2 and MB3) using a paired *t* test to determine whether there are any significant differences between methods in the number of subjects needed to detect a between-group change.

## Results

### Visual comparison

**Fig. 2:**
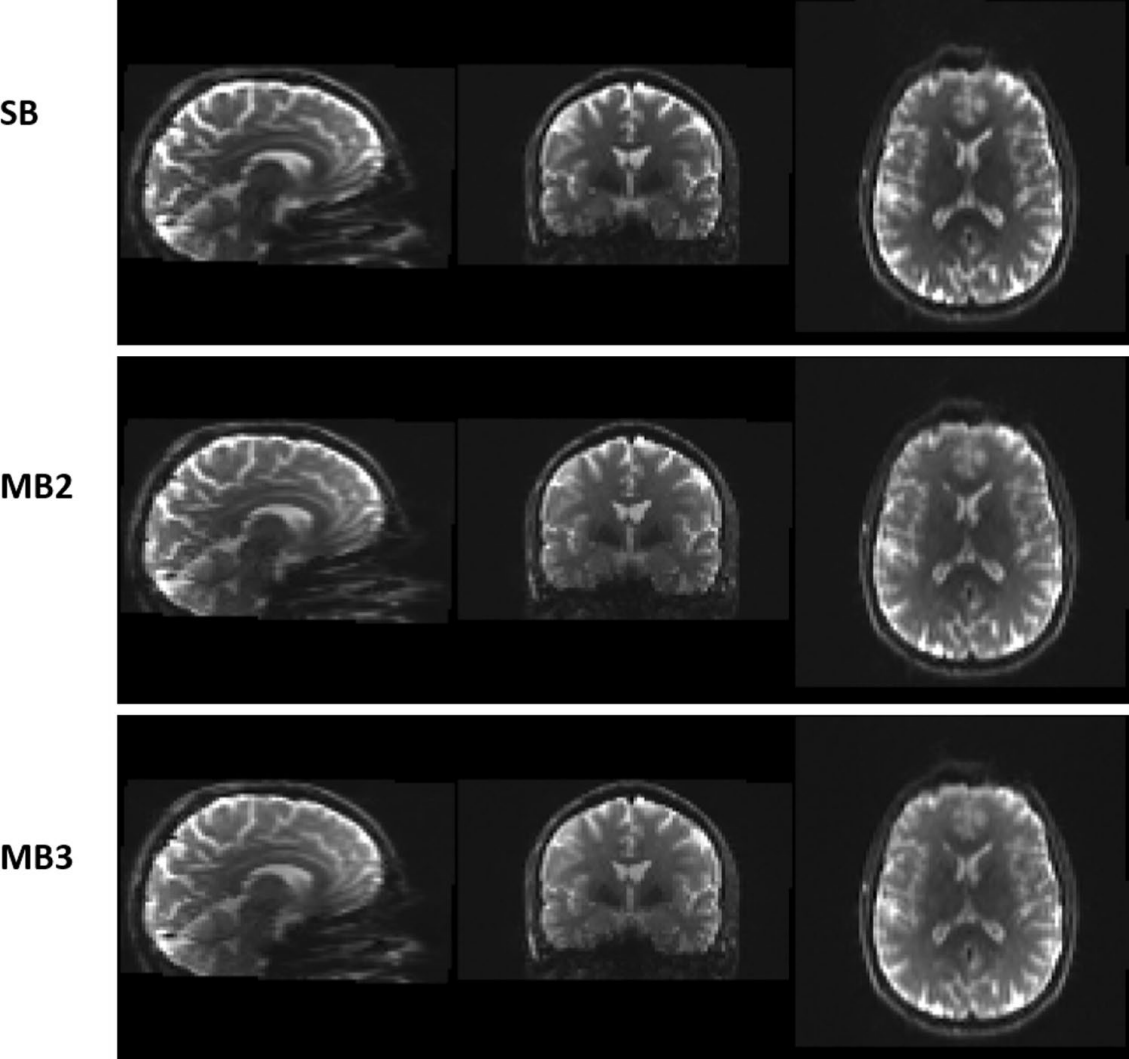
Representative co-registered slices from *b* = 0 images from *SB* singleband, *MB2* multiband factor 2 and *MB3* multiband factor 3 data taken from subject one during visit one

Prior to performing NODDI fitting, a visual inspection has been carried out on all the pre-processed diffusion data. Figure 2 shows co-registered slices from SB, MB2 and MB3 images taken from a representative subject during visit 1; the figure illustrates there are no appreciable differences in artefacts or image quality.

### SNR

**Fig. 3:**
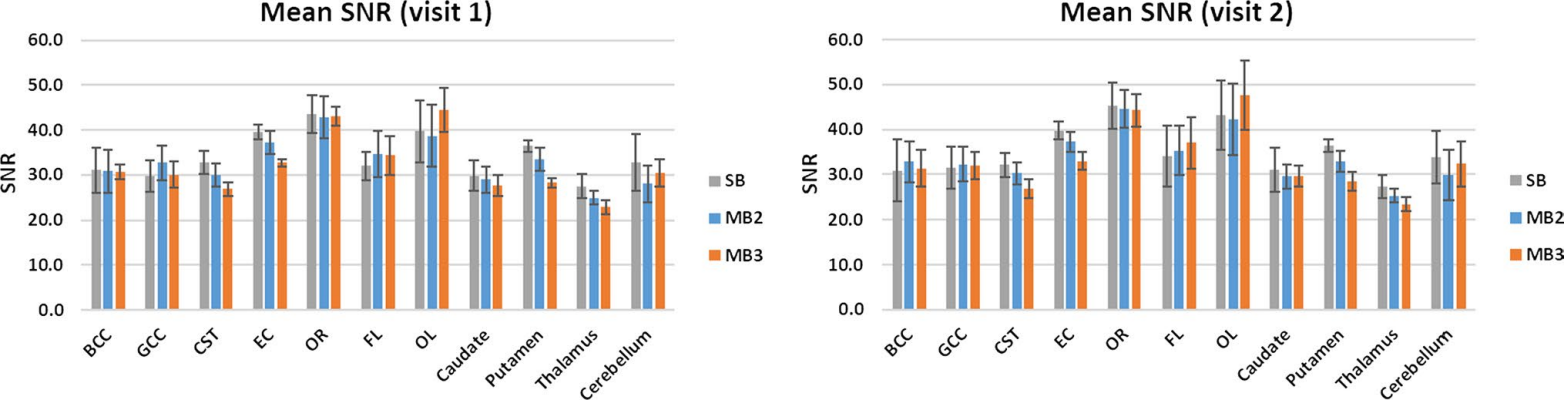
Signal to noise ratio (SNR) for data acquired using, *SB* singleband, *MB2* multiband factor 2 and *MB3* multiband factor 3. SNR is reported for the following regions of interest: *BCC* body of corpus callosum, *GCC* genu of corpus callosum, *CST* corticospinal tracts, *EC* external capsules, *OR* optic radiation, *FL* frontal lobe, *OL* occipital lobe, caudate, putamen thalamus and cerebellum

Figure 3 shows the average SNR for each ROI for all the tested acquisitions (averaged across subjects, SD across subjects is shown as error bars). As expected, the multiband factor has the overall effect of reducing SNR, particularly when increasing the acceleration factor from 2 to 3, but not significantly.

Inspecting the voxel-wise SNR (see the supplementary material), we have observed that the SNR was more uniform across the brain in SB; whereas in MB SNR had a greater dependence on tissue-type and location with lowest values recorded in the deeper areas of the brain. Furthermore, comparing mean SNR for SB, MB2 and MB3, has revealed a significant decrease in SNR, particularly in white matter ROIs and deep gray matter ROIs as the MB factor increases (*p*<0.05).

### Signal variability across diffusion weighting directions

**Fig. 4:**
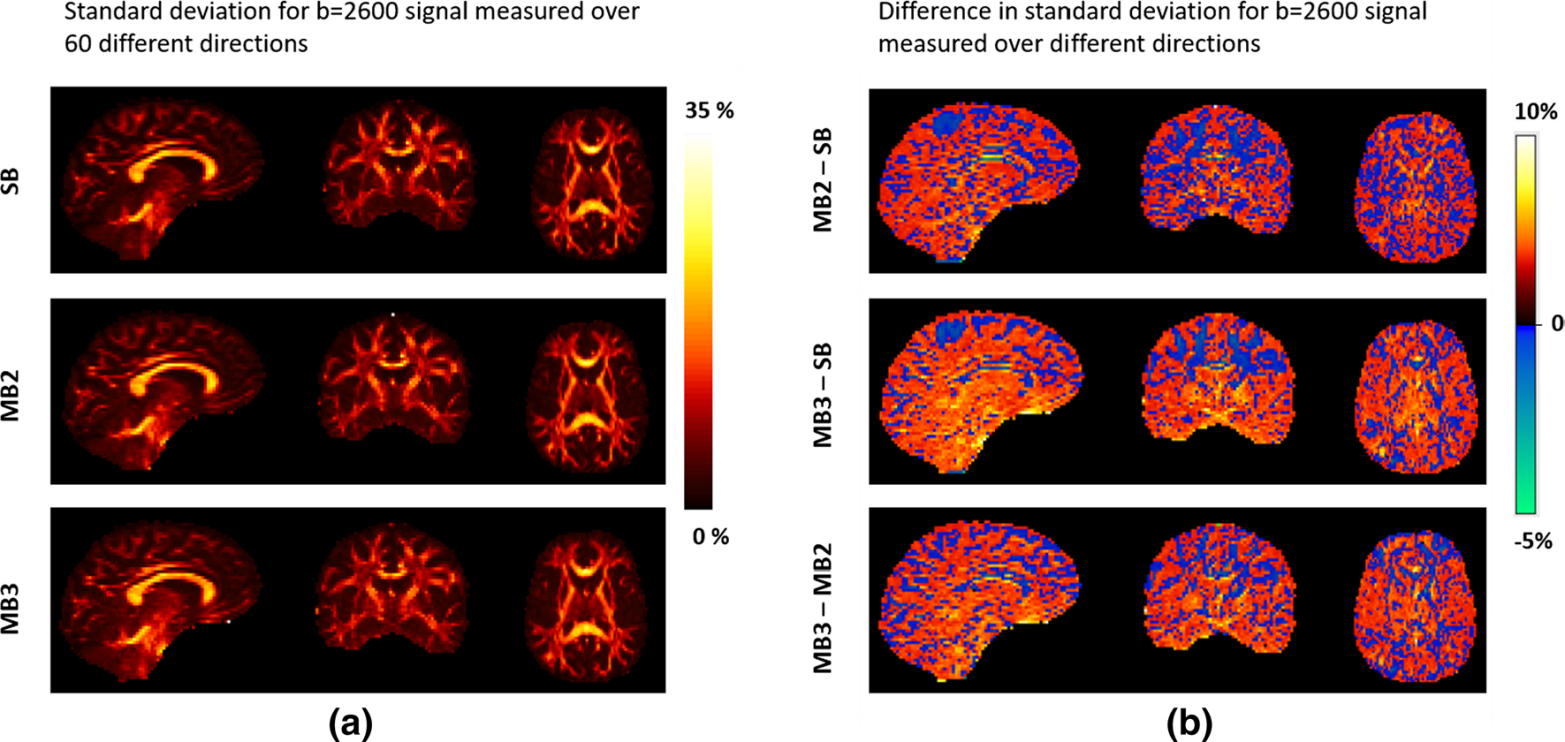
**a** Variability between diffusion signal between *b* = 2600 data points measured over 60 different directions from SB, MB2 and MB3. And (**b**) difference in signal variability between each pair of acquisitions SB, MB2 and MB3. *SB* singleband, *MB2* multiband factor 2 and *MB3* multiband factor 3 (data taken from subject one during visit one)

Figure 4 shows the variability between diffusion signal between *b* = 2600 data points measured over 60 different directions, from a representative subject in visit 1. The results show that the variability was increased in GM and slightly decreased in WM, as the MB factor increased.

### Variability between subjects and between visits

**Fig. 5:**
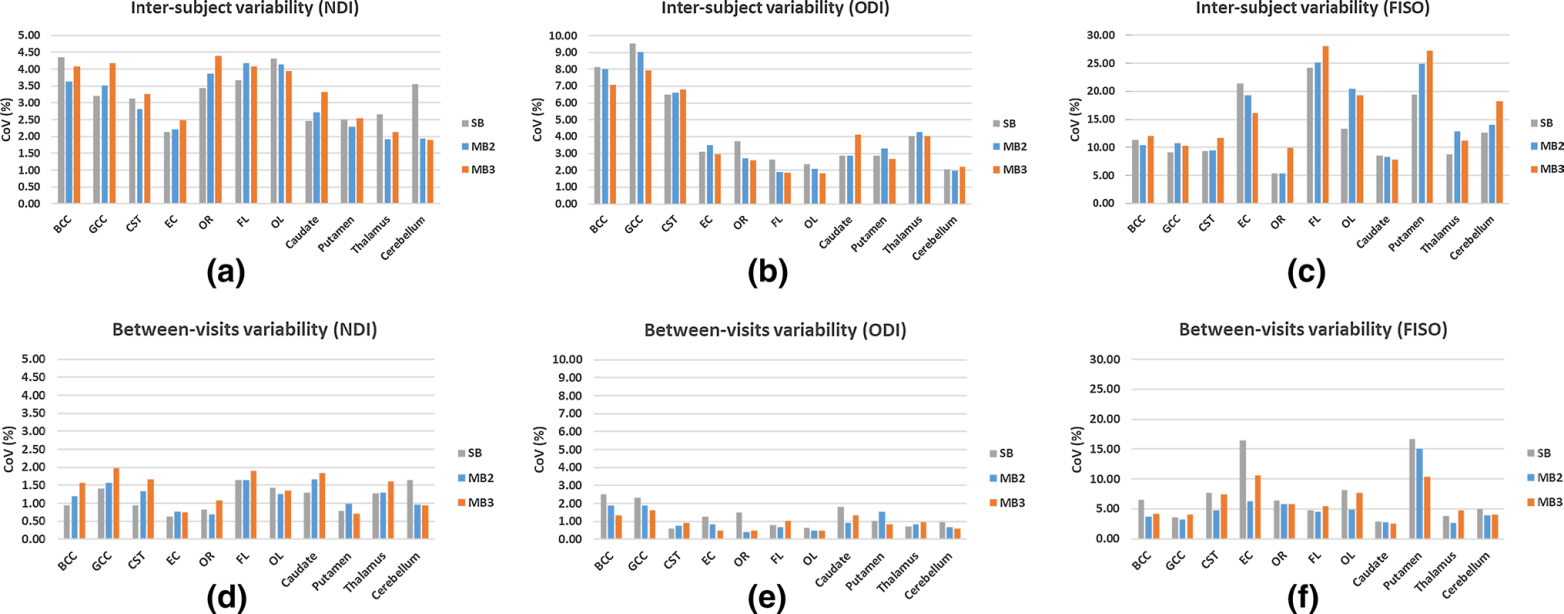
Inter-subject and between-visits variability analysis of NODDI parameters estimated from diffusion data acquired. Variability measure is calculated using Coefficients of Variation (CoV) on: *BCC* body of corpus callosum, *GCC* genu of corpus callosum, *CST* corticospinal tracts, *EC* external capsules, *OR* optic radiation, *FL* frontal lobe, *OL* occipital lobe, caudate, putamen thalamus and cerebellum. *SB* singleband, *MB2* multiband factor 2 and *MB3* multiband factor 3; *NDI* neurite density index, *ODI* orientation dispersion index and FISO = CSF volume fraction

CoVs for NDI, FISO and ODI are shown in Fig. 5 (see Table 1S in the supplementary material for a tabular breakdown of these CoV measures)**.** The results show that between subjects CoV measurements (Fig. 5a–c) are approximately 2 times higher than between visits CoV measurements (Fig. 5d–f). NDI exhibited the lowest variation of all NODDI parameters between visits and subjects with CoV<2% and <4.5%, respectively. The highest CoVs for NDI were: between visits 1.64% (SB), 1.67% (MB2) and 1.97% (MB3); and between subjects 4.36% (SB), 4.17% (MB2) and 4.40% (MB3). Similarly, ODI showed a low variability between scan visits for all ROIs and regardless of the acceleration factor with the following highest CoVs: 2.50% (SB), 1.88% (MB2) and 1.63% (MB3). On the other hand, ODI showed relatively larger between-subjects variability (CoV between 6.5% and 10%) in the white matter regions with prevalence of tightly packed parallel fibres (e.g. GCC, BCC and CST) than the gray matter regions. FISO shows the largest variation with the following highest CoVs: between visits 16.69% (SB), 15.09% (MB2) and 10.58% (MB3); and between subjects 24.15% (SB), 25.17% (MB2) and 28.06% (MB3).

There is greater variation between subjects than between visits for all NODDI parameters. This is expected because a normal physiological variation within the brain microstructure is known to give rise to variability in quantitative MR measurements in general, even in homogenous groups like those studied here [39].

### Reproducibility between visits

**Fig. 6:**
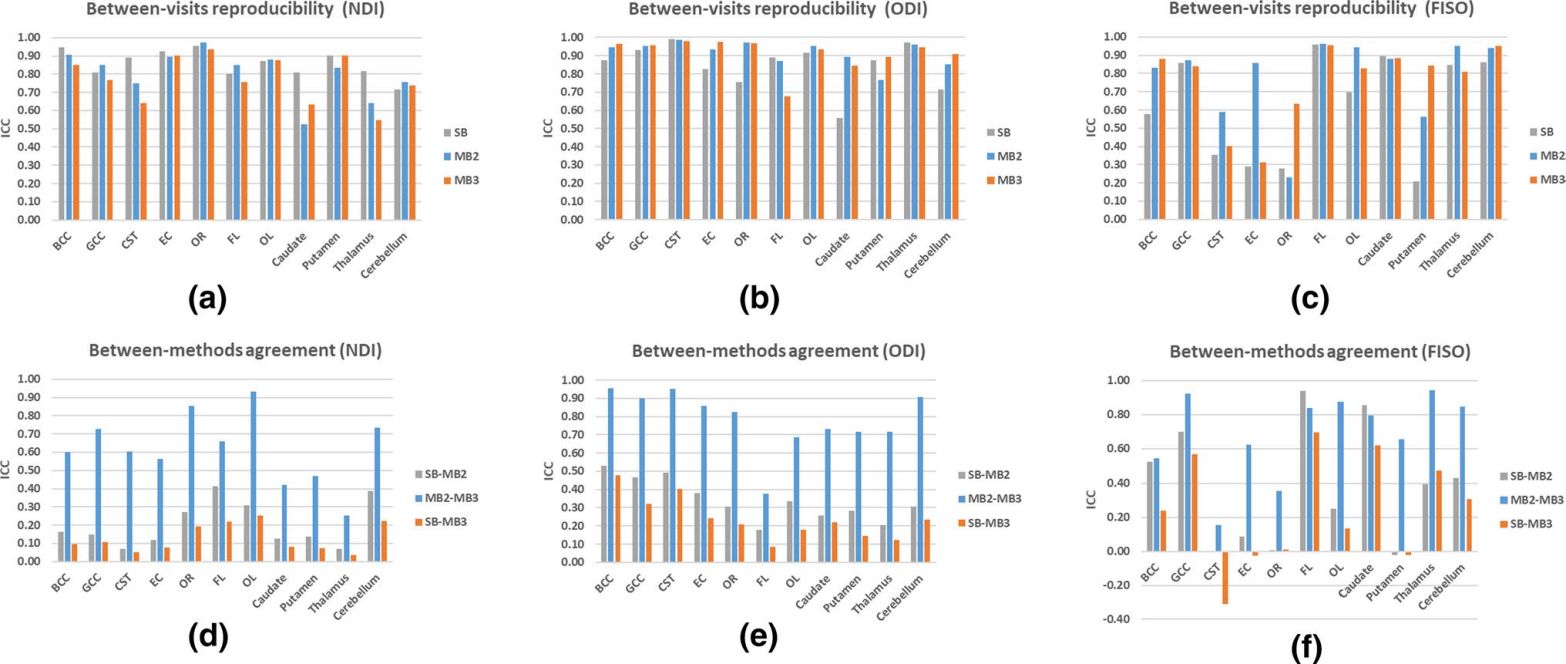
Visualisation of between-visits reproducibility for SB, MB2 and MB3; and between-methods agreement within the first visit to estimate NODDI parameters. This is measured using intra-class correlation analysis for the following regions of interest: *BCC* body of corpus callosum, *GCC* genu of corpus callosum, *CST* corticospinal tracts, *EC* external capsules, *OR* optic radiation, *FL* frontal lobe, *OL* occipital lobe, caudate, putamen thalamus and cerebellum. *SB* singleband, *MB2* multiband factor 2 and *MB3* multiband factor 3; *NDI* neurite density index, *ODI* orientation dispersion index and FISO = CSF volume fraction

Figure 6a–c shows the ICC estimates between visit 1 and visit 2 for NDI, FISO and ODI calculated separately for SB, MB2 and MB3 acquisition methods (see Table 2S in the supplementary material for a tabular breakdown of these ICC measures). The results demonstrate that the ICC of NODDI metrics collected with SB acquisition consistently showed good to excellent reproducibility between visits for NDI for all ROIs with ICC ranging between 0.72 and 0.95. Whereas MB2 and MB3 exhibited moderate to excellent reproducibility for all ROIs with recorded ICCs between 0.53 and 0.97 for MB2, and between 0.55 and 0.94 for MB3. ODI, on the other hand, produced good to excellent reproducibility which appears to be independent of the MB acceleration factor, for most regions (ICC between 0.75 and 0.99) except the caudate, cerebellum and frontal lobe. These three regions produced moderate ICCs for ODI of 0.56 (SB in the caudate), 0.71 (SB in the cerebellum) and 0.68 (MB3 in the frontal lobe). Finally, when examining FISO’s reproducibility between the visits, we observed smaller ICC (≤ 0.58) for half of the ROIs particularly in SB data. Unlike SB and MB3 data, MB2 produced moderate to excellent ICC values in FISO, except in the optic radiation. This variability in the performance of FISO renders it the least reliable of the NODDI measures with inconsistent or poor reproducibility for several regions.

### Agreement between acquisition methods

We compared the data acquired between each two methods of different MB acceleration factors from visit 1 by means of ICC as well as Bland–Altman analysis. The results from the ICC analysis are reported in Fig. 6d–f. The ICC measurements showed poor agreement between SB and MB2 and between SB and MB3 for all parameters with ICC < 0.5 for most regions. Comparing MB2 with MB3 showed relatively better agreement, than comparisons with SB, particularly in ODI which produced ICC > 0.7 for several regions.

**Fig. 7:**
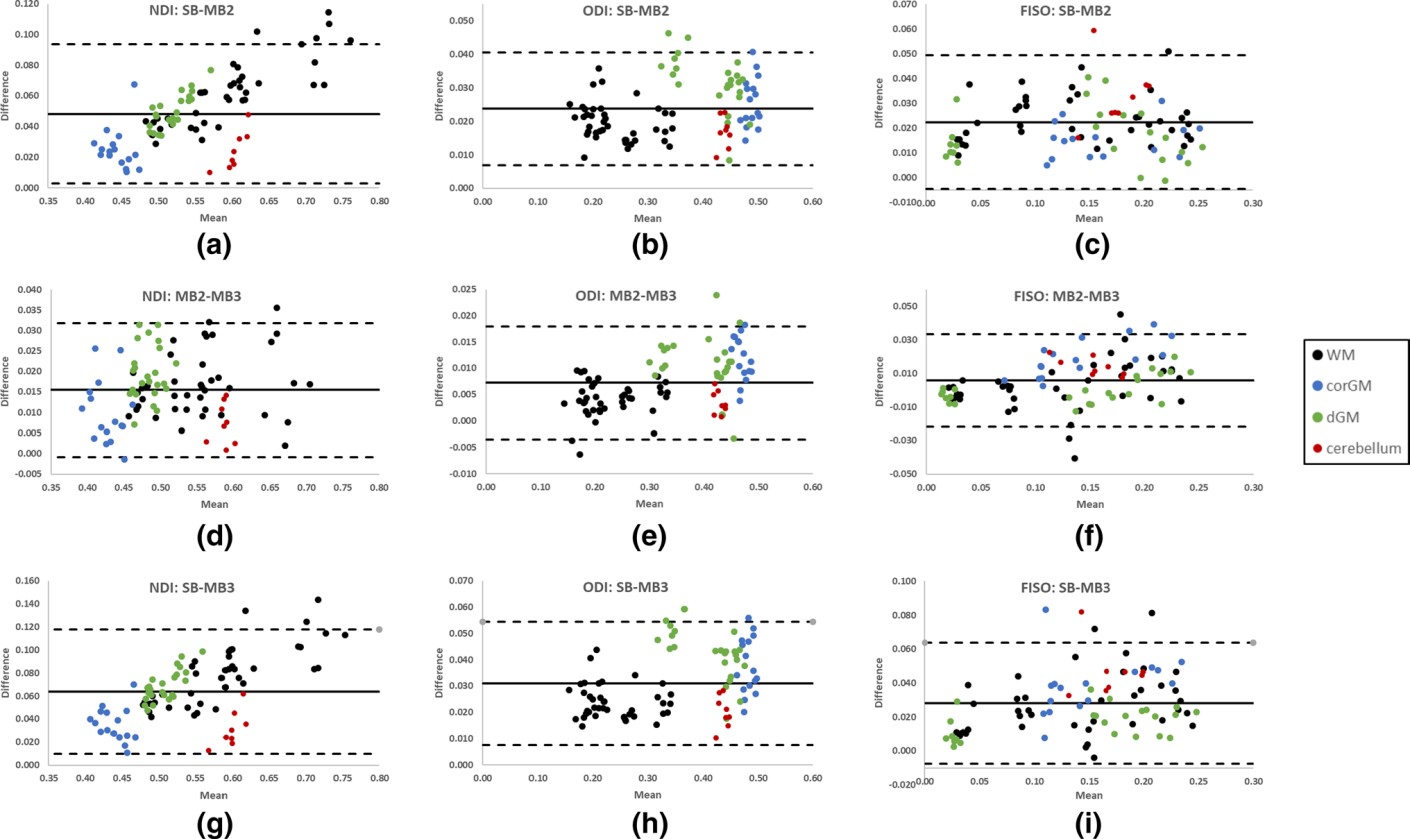
Bland–Altman plot of SB vs MB2 (**a**, **b**, **c**),MB2 vs MB3 (**d**, **e**, **f**) and SB vs MB3 (**g**, **h**, **i**) to estimate ODI, FISO and NDI measures (from visit 1) in all subjects for the following ROIs: *BCC* body of corpus callosum, *GCC* genu of corpus callosum, *CST* corticospinal tracts, *EC* external capsules, *OR* optic radiation, *FL* frontal lobe, *OL* occipital lobe, caudate, putamen thalamus and cerebellum. *SB* singleband, *MB2* multiband factor 2 and *MB3* multiband factor 3. (Datapoints’ colour represents different tissues: black for white matter (WM), blue for cortical gray matter (corGM), green for deep gray matter (dGM) and red for the cerebellum)

In order to examine the extent of agreement between methods, Bland–Altman plots for data acquired in visit 1 were produced as shown in Fig. 7. Comparing SB with MB2 revealed a systematic bias with parameters estimated from SB data being consistently higher as compared to those from MB2 data (Fig. 7a–c), with a bias of 0.048 in NDI, 0.024 in ODI and 0.022 in FISO. A similar trend was observed when comparing SB with MB3 (Fig. 7g–i), with a reported bias of 0.064 in NDI, 0.031 in ODI and 0.028 in FISO. On closer inspection, the systematic bias can be seen to be correlated with the MB factor, with both ODI and NDI decreasing as the MB factor increases from 1 to 3 (SB, > MB2, > MB3). Comparing MB2 with MB3 showed a much smaller bias, (0.015 in NDI, 0.007 in ODI and 0.006 in FISO), with MB2 exhibiting higher values (Fig. 7d–f).

Interestingly, the difference in NDI measures between SB and MB2 appeared to be dependent on the NDI value itself, Fig. 7a, (correlation coefficient=0.71, *p* value<0.001, slope=0.20) and it is not clear what is driving this dependency. A similar relationship is detected between SB and MB3, Fig. 7g (correlation coefficient=0.63, *p* value<0.001, slope=0.22). It should be noted that repeating the Bland–Altman analysis on data collected in visit 2 confirms the observations made using the first visit’s data (see Fig. 2S in Supplementary Material).

**Fig. 8:**
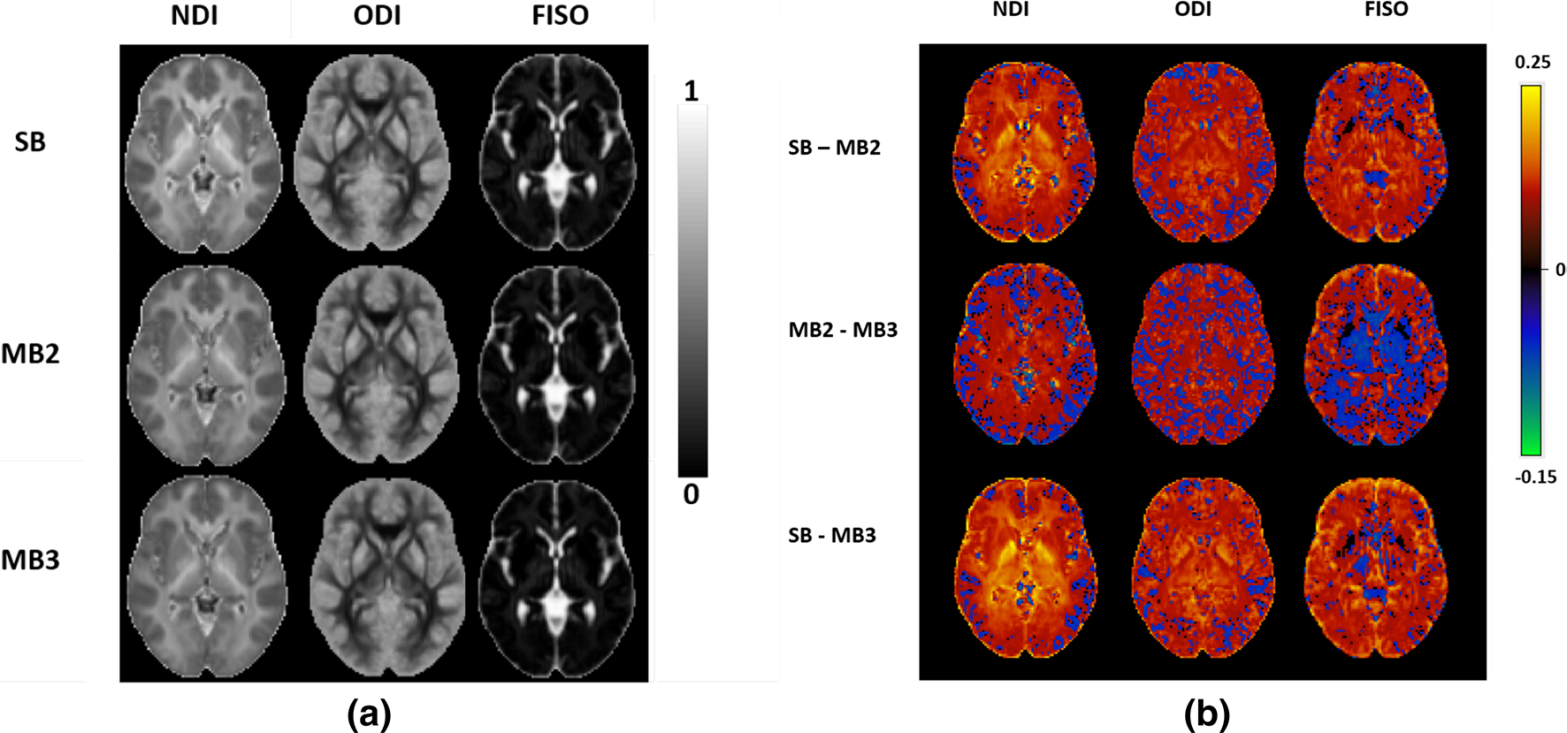
**a** Average images for ODI, FISO and NDI maps (from visit 1 across subjects) for SB, MB2 and MB3 data. And (**b**) difference images for ODI, FISO and NDI maps between SB vs MB2, MB2 vs MB3 and SB vs MB3. *SB* singleband, *MB2* multiband factor 2 and *MB3* multiband factor 3

The mean parameter maps across subjects for each NODDI metric for SB, MB2 and MB3 help to visualise any systematic bias reported in Bland–Altman analysis (Fig. 8a). A strong similarity is seen for all three methods and all NODDI parameter maps have maintained similar contrast between tissues, with no obvious differences in artefact. Subtle underlying differences between the methods are shown in the difference maps (Fig. 8b), where the difference in NDI is dependent on the mean, as expected from the Bland–Altman analysis, and appear to be concentrated in the centre of the FOV, which can be attributed to effects of MB reconstruction or to the shorter TR in MB. In fact, the pattern follows the same pattern of the reduced SNR in those areas (see Supplementary Fig. 1S). On the other hand, differences in ODI and FISO look less dependent on the location within the brain and MB factor, with a relatively more uniform distribution of positive and negative differences between MB2 and MB3, which is expected given these two techniques show the closest agreement.

### Power and sample size calculations

Since between visits variability is lower than the inter-subject variability, within-subject changes can be detected with group sizes typical of small-to-moderate research studies. We quantified the sample sizes required for detecting reductions of 5% and 10% in all three NODDI metrics (Tables 1, 2, 3. Our results have shown that for NDI, the required sample size for detecting a between-group change is comparable for all methods independent of the MB acceleration factor (*N* ≤ 18 for group differences of 5%, Table 1). In order to detect a similar group differences in ODI, particularly in tightly packed white matter regions, greater sample sizes are required (between 37 and 78, Table 2); this is due to the larger variability between subjects in these areas as reported earlier in Fig. 5. The sample sizes required to detect group differences in FISO are considerably larger (Table 3); this is due to the larger variability between subjects for this parameter, particularly with MB. It is worth noting that the choice of NODDI measure to be studied and the ROI will depend on the type and site of pathology.

**Table 1:** Required sample size (per group) for between subject comparisons of mean NDI for following ROIs

	SB (NDI)		MB2 (NDI)		MB3 (NDI)		SB required *N* for effect size of		MB2 required *N* for effect size of		MB3 required *N* for effect size of	
	MEAN	SD	MEAN	SD	MEAN	SD	5%	10%	5%	10%	5%	10%
BCC	0.643	0.028	0.572	0.021	0.549	0.022	17	6	13	5	15	5
GCC	0.635	0.020	0.569	0.020	0.552	0.023	10	4	12	4	16	5
CST	0.768	0.024	0.678	0.019	0.659	0.021	10	4	8	4	10	4
EC	0.516	0.011	0.478	0.011	0.465	0.012	6	3	6	3	7	3
OR	0.574	0.020	0.531	0.021	0.519	0.023	11	4	14	5	18	6
FL	0.450	0.017	0.423	0.018	0.408	0.017	13	5	16	5	16	5
OL	0.464	0.020	0.441	0.018	0.435	0.017	17	6	16	5	15	5
Caudate	0.532	0.013	0.488	0.013	0.467	0.015	7	3	8	3	11	4
Putamen	0.520	0.013	0.479	0.011	0.463	0.012	7	3	6	3	7	3
Thalamus	0.575	0.015	0.512	0.010	0.491	0.010	8	3	5	3	6	3
Cerebellum	0.615	0.022	0.591	0.011	0.583	0.011	12	4	5	3	5	3

*BCC* body of corpus callosum, *GCC* genu of corpus callosum, *CST* corticospinal tracts, *EC* external capsules, *OR* optic radiation, *FL* frontal lobe, *OL* occipital lobe, caudate, putamen, thalamus and cerebellum

**Table 2:** Required sample size (per group) for between subject comparisons of mean ODI for following ROIs

	SB (ODI)		MB2 (ODI)		MB3 (ODI)		SB required *N* for effect size of		MB2 required *N* for effect size of		MB3 required *N* for effect size of	
	MEAN	SD	MEAN	SD	MEAN	SD	5%	10%	5%	10%	5%	10%
BCC	0.216	0.018	0.196	0.016	0.194	0.014	57	15	55	15	44	12
GCC	0.198	0.019	0.173	0.016	0.168	0.013	78	21	70	19	54	15
CST	0.217	0.014	0.198	0.013	0.194	0.013	37	10	38	11	41	11
EC	0.341	0.011	0.323	0.011	0.318	0.009	10	4	12	4	9	4
OR	0.276	0.010	0.260	0.007	0.255	0.007	13	5	8	3	7	3
FL	0.502	0.013	0.471	0.009	0.455	0.008	8	3	5	3	5	3
OL	0.501	0.012	0.480	0.010	0.472	0.009	6	3	5	3	5	3
Caudate	0.463	0.013	0.437	0.013	0.427	0.018	8	4	9	4	16	5
Putamen	0.476	0.014	0.446	0.015	0.436	0.012	9	4	11	4	8	3
Thalamus	0.368	0.015	0.330	0.014	0.318	0.013	15	5	17	6	15	5
Cerebellum	0.447	0.009	0.430	0.008	0.427	0.009	5	3	5	3	6	3

*BCC* body of corpus callosum, *GCC* genu of corpus callosum, *CST* corticospinal tracts, *EC* external capsules, *OR* optic radiation, *FL* frontal lobe, *OL* occipital lobe, caudate, putamen, thalamus and cerebellum

**Table 3:** Required sample size (per group) for between subject comparisons of mean FISO for following ROIs

	SB (FISO)		MB2 (FISO)		MB3 (FISO)		SB required *N* for effect size of		MB2 required *N* for effect size of		MB3 required *N* for effect size of	
	MEAN	SD	MEAN	SD	MEAN	SD	5%	10%	5%	10%	5%	10%
BCC	0.221	0.025	0.196	0.020	0.173	0.021	109	28	92	24	122	32
GCC	0.234	0.021	0.215	0.023	0.210	0.022	70	19	98	26	90	24
CST	0.159	0.015	0.126	0.012	0.137	0.016	75	20	76	20	117	30
EC	0.044	0.009	0.026	0.005	0.028	0.005	385	98	315	79	220	56
OR	0.102	0.005	0.074	0.004	0.076	0.008	25	8	25	8	85	22
FL	0.204	0.049	0.188	0.047	0.160	0.045	492	124	532	135	665	167
OL	0.137	0.018	0.115	0.023	0.104	0.020	149	39	354	89	315	79
Caudate	0.228	0.019	0.219	0.018	0.210	0.016	63	17	59	16	52	14
Putamen	0.032	0.006	0.018	0.004	0.021	0.006	315	80	522	132	622	157
Thalamus	0.180	0.016	0.152	0.020	0.157	0.018	66	18	140	36	107	28
Cerebellum	0.193	0.024	0.160	0.022	0.146	0.027	135	35	166	43	279	71

*BCC* body of corpus callosum, *GCC* genu of corpus callosum, *CST* corticospinal tracts, *EC* external capsules, *OR* optic radiation, *FL* frontal lobe, *OL* occipital lobe, caudate, putamen, thalamus and cerebellum

Finally, there was no significant differences (*p*>0.05) in the group sizes required to detect between-group changes for the three acquisition methods

## Discussion

We have studied the reproducibility of NODDI parameters from datasets acquired using both conventional SB and the faster MB EPI acquisition on a 3 T MRI scanner. When comparing the data acquired with the same multiband factor, we found that NODDI parameters, particularly NDI and ODI, are highly reproducible between subjects and visits, while the parameter that showed the worst reproducibility is FISO.

Both NDI and ODI exhibited moderate to high within-subject reproducibility across the sessions for both SB and MB data, with CoV falling within an acceptable range for all regions (CoV < 2.5%), comparable to those obtained in similar studies for DTI parameters [40, 41]. This suggests their potential to be used for exploring individual differences and potentially for clinical applications. FISO, however, was highly varied, particularly across these healthy subjects where CoV values were typically between 5.31% and 28.06% for all ROIs, which raises questions over its reliability and relevance as a useful biomarker. Indeed, the accuracy of FISO has been questioned before, with reports showing that NODDI tends to overestimate FISO because a single T2 is assumed for all voxel compartments [9] and also due to the contribution of perfusion to the overall signal [42], rendering it the least reliable parameter. Yet these observations alone do not account for the poor reproducibility observed here. A possible explanation is that the hierarchical nature of the NODDI model increases the likelihood of any deviation of the signal being disproportionally attributed to the estimation of FISO, rather than NDI or ODI.

Although ODI showed a low variability between visits for all ROIs regardless of the MB acceleration factor, it showed a relatively large between-subjects variability in tightly packed white matter tracts (e.g. BCC, GCC and CST). This could be explained by the fact that ODI is close to zero in tightly packed white matter regions and thus small variations (including those introduced by noise) result in high percentage change.

Comparing NODDI metrics acquired with different multiband factor (SB, MB2, MB3) revealed a systematic bias across all NODDI metrics. This was highlighted by the Bland–Altman analysis that also demonstrated that the magnitude of the bias increases for all parameters (with respect to SB) as the MB factor increases. A number of factors may be responsible for this effect. The reduction in TR achieved with increasing MB factor will result in a reduction in SNR due to increased T1 weighting. This is more evident in regions with long T1, such as the gray matter. Although, we observe a non-negligible variation in SNR with anatomical location, leading to some regions having slightly higher SNR in MB data. This is unexpected and unlikely to be due to reduced subject movements during shorter acquisition times. Furthermore, it is known that the SNR in MB images is not solely impacted by lower TR, but also the MB and GRAPPA reconstruction. Indeed, SNR is dependent on the geometric arrangement of the parallel imaging receiver coils; the *g* factor [24]. As a result, non-uniform SNR is observed with some ROIs exhibiting worse SNR than others [26, 28]. In addition, using GRAPPA will compound this effect resulting in the reduction in SNR across most ROIs studied here. Our work reveals that NDI exhibits the largest bias with respect to SB as MB factor is increased. A likely driver for this reduction in NDI is the reduced SNR; previous studies have identified that NDI is particularly sensitive to noise and is known to decrease as SNR drops a reported by Hutchinson et al. [43]. Furthermore, the greatest negative bias in NDI are observed in WM and deep GM regions which were previously shown to exhibit the strongest decreases in SNR.

The systematic differences in ODI were slightly positively correlated with the ODI value itself and the estimated ODI decreased with the increase of MB factor. It is not clear what is driving this bias and no obvious correlation was observed between the spatial distribution of the differences in the standard deviation of the raw signal between SB and MB and the difference in ODI value.

Finally, the power calculation suggested that the number of participants required to detect an effect depends on the specific parameter of interest (NDI, ODI and FISO) and on the anatomical area under study. In addition, the number of subjects needed to detect a between-group change is not significantly different between methods. As previously mentioned, FISO has the worst reproducibility and this is reflected in the higher sample size requirements. FISO is also particularly sensitive to the ROI (location and size); this could be due to partial volume effects with CSF adding to the variability in this metric.

This work has identified some important considerations for incorporating MB into NODDI studies. We have also suggested a number of factors that are potential drivers of the systematic bias introduced by changing the multiband factor and it is clear that further work is required to investigate the effects fully. However, this lies beyond the scope of this study.

## Conclusions

We can conclude that reliable and reproducible NDI and ODI measures are provided by all acquisition methods studied here (SB, MB2 or MB3). However, we also observed that the acquisition method introduces a significant bias to these parameters such that a direct comparison between NODDI indices acquired with SB, MB2 and MB3 is not trivial. This is an extremely important consideration for studies that seek to compare NODDI data acquired using a different MB factor (e.g. in multicentre studies or when comparing with the previous studies in the literature). The parameter that showed the worst reproducibility is FISO. This work also shows that the attractive time-saving benefits of MB acquisition does not come with the penalty of requiring larger sample sizes when making group comparisons. Indeed, the greater time-efficiency of MB acquisition, could be exploited to collect a richer diffusion-weighted data set (additional diffusion directions or diffusion weightings) to improve the reliability of the NODDI parameters further. These findings must be taken into consideration when planning future studies.

## Supplementary Information

Below is the link to the electronic supplementary material.

Supplementary file1 Table 1S: Inter-subject (a) and between-visits (b) variability analysis of NODDI parameters estimated from diffusion data acquired. Variability measure is calculated using Coefficients of Variation (CoV) on: Body of Corpus Callosum BCC, Genu of Corpus Callosum GCC, Corticospinal Tracts CST, External Capsules EC, Optic Radiation OR, Frontal Lobe FL, Occipital Lobe OL, Caudate, Putamen Thalamus and Cerebellum. SB = Singleband, MB2 = Multiband factor 2 and MB3 = Multiband factor 3; NDI = neurite density index, ODI = orientation dispersion index and FISO = CSF volume fraction. Table 2S: Between-visits reproducibility (a) for SB, MB2 and MB3; and between-methods agreement (b) within the first visit to estimate NODDI parameters. This is measured using intra-class correlation analysis for the following regions of interest: Body of Corpus Callosum BCC, Genu of Corpus Callosum GCC, Corticospinal Tracts CST, External Capsules EC, Optic Radiation OR, Frontal Lobe FL, Occipital Lobe OL, Caudate, Putamen Thalamus and Cerebellum. SB = Singleband, MB2 = Multiband factor 2 and MB3 = Multiband factor 3; NDI = neurite density index, ODI = orientation dispersion index and FISO = CSF volume fraction (DOCX 29 KB)

Supplementary file2 Fig. 1S: Signal to noise ratio in diffusion signal for data acquired using Singleband EPI (SB) sequence, Multiband EPI factor 2 (MB2) and Multiband EPI factor 3 (MB3) (data taken from subject one during visit one). Fig. 2S: Bland-Altman plot of SB vs MB2 (a-c),MB2 vs MB3 (d-f), and SB vs MB3 (g-i) to estimate ODI, FISO and NDI measures (from visit 2) in all subjects for the following ROIs: Body of Corpus Callosum BCC, Genu of Corpus Callosum GCC, Corticospinal Tracts CST, External Capsules EC, Optic Radiation OR, Frontal Lobe FL, Occipital Lobe OL, Caudate, Putamen Thalamus and Cerebellum. SB = Singleband, MB2 = Multiband factor 2 and MB3 = Multiband factor 3. (Datapoints’ colour represents different tissues: black for white matter (WM), blue for cortical gray matter (corGM), green for deep gray matter (dGM) and red for the cerebellum). (PPTX 888 KB)
