# Supplementary material for: Comparing multiband and singleband EPI in NODDI at 3 T: what are the implications for reproducibility and study sample sizes?
Source: MAGMA. 2020 Dec 14;34(4):499–511. doi: 10.1007/s10334-020-00897-7 (PMC8338814; doi:10.1007/s10334-020-00897-7)
Supplement: Supplementary file 1 — Supplementary file1 Table 1S: Inter-subject (a) and between-visits (b) variability analysis of NODDI parameters estimated from diffusion data acquired. Variability measure is calculated using Coefficients of Variation (CoV) on: Body of Corpus Callosum BCC, Genu of Corpus Callosum GCC, Corticospinal Tracts CST, External Capsules EC, Optic Radiation OR, Frontal Lobe FL, Occipital Lobe OL, Caudate, Putamen Thalamus and Cerebellum. SB = Singleband, MB2 = Multiband factor 2 and MB3 = Multiband factor 3; NDI = neurite density index, ODI = orientation dispersion index and FISO = CSF volume fraction. Table 2S: Between-visits reproducibility (a) for SB, MB2 and MB3; and between-methods agreement (b) within the first visit to estimate NODDI parameters. This is measured using intra-class correlation analysis for the following regions of interest: Body of Corpus Callosum BCC, Genu of Corpus Callosum GCC, Corticospinal Tracts CST, External Capsules EC, Optic Radiation OR, Frontal Lobe FL, Occipital Lobe OL, Caudate, Putamen Thalamus and Cerebellum. SB = Singleband, MB2 = Multiband factor 2 and MB3 = Multiband factor 3; NDI = neurite density index, ODI = orientation dispersion index and FISO = CSF volume fraction (DOCX 29 KB) [file 10334_2020_897_MOESM1_ESM.docx]

**Table 1S**

| **a - Inter-subject variability** | | | | | | | | | |
| --- | --- | --- | --- | --- | --- | --- | --- | --- | --- |
|  | **NDI** | | | **ODI** | | | **FISO** | | |
|  | **SB** | **MB2** | **MB3** | **SB** | **MB2** | **MB3** | **SB** | **MB2** | **MB3** |
| **BCC** | 4.36 | 3.63 | 4.08 | 8.11 | 8.01 | 7.09 | 11.29 | 10.37 | 11.99 |
| **GCC** | 3.20 | 3.50 | 4.17 | 9.52 | 9.03 | 7.94 | 9.04 | 10.71 | 10.28 |
| **CST** | 3.12 | 2.81 | 3.25 | 6.48 | 6.60 | 6.82 | 9.35 | 9.39 | 11.70 |
| **EC** | 2.14 | 2.21 | 2.48 | 3.10 | 3.51 | 2.95 | 21.41 | 19.28 | 16.11 |
| **OR** | 3.43 | 3.87 | 4.40 | 3.71 | 2.71 | 2.59 | 5.31 | 5.34 | 9.96 |
| **FL** | 3.67 | 4.17 | 4.08 | 2.65 | 1.89 | 1.86 | 24.15 | 25.17 | 28.06 |
| **OL** | 4.31 | 4.14 | 3.93 | 2.37 | 2.08 | 1.81 | 13.28 | 20.45 | 19.28 |
| **Caudate** | 2.47 | 2.71 | 3.32 | 2.85 | 2.87 | 4.13 | 8.55 | 8.31 | 7.78 |
| **Putamen** | 2.51 | 2.29 | 2.54 | 2.87 | 3.28 | 2.66 | 19.33 | 24.85 | 27.22 |
| **Thalamus** | 2.65 | 1.92 | 2.13 | 4.06 | 4.26 | 4.02 | 8.76 | 12.82 | 11.19 |
| **Cerebellum** | 3.56 | 1.94 | 1.91 | 2.03 | 1.96 | 2.20 | 12.61 | 14.00 | 18.20 |
| **b - Between-visits variability** | | | | | | | | | |
|  | **NDI** | | | **ODI** | | | **FISO** | | |
|  | **SB** | **MB2** | **MB3** | **SB** | **MB2** | **MB3** | **SB** | **MB2** | **MB3** |
| **BCC** | 0.95 | 1.19 | 1.58 | 2.50 | 1.87 | 1.35 | 6.47 | 3.73 | 4.11 |
| **GCC** | 1.41 | 1.57 | 1.97 | 2.33 | 1.88 | 1.63 | 3.62 | 3.23 | 4.04 |
| **CST** | 0.94 | 1.34 | 1.66 | 0.60 | 0.76 | 0.91 | 7.67 | 4.75 | 7.49 |
| **EC** | 0.63 | 0.76 | 0.76 | 1.26 | 0.82 | 0.47 | 16.44 | 6.23 | 10.58 |
| **OR** | 0.82 | 0.69 | 1.09 | 1.50 | 0.42 | 0.47 | 6.33 | 5.77 | 5.77 |
| **FL** | 1.64 | 1.65 | 1.91 | 0.79 | 0.66 | 1.04 | 4.74 | 4.53 | 5.46 |
| **OL** | 1.44 | 1.26 | 1.35 | 0.66 | 0.47 | 0.49 | 8.15 | 4.86 | 7.64 |
| **Caudate** | 1.30 | 1.67 | 1.83 | 1.80 | 0.93 | 1.35 | 2.91 | 2.75 | 2.48 |
| **Putamen** | 0.79 | 0.98 | 0.72 | 1.02 | 1.55 | 0.83 | 16.69 | 15.09 | 10.38 |
| **Thalamus** | 1.27 | 1.29 | 1.60 | 0.73 | 0.82 | 0.94 | 3.78 | 2.67 | 4.79 |
| **Cerebellum** | 1.64 | 0.96 | 0.94 | 0.96 | 0.69 | 0.59 | 5.02 | 3.90 | 4.10 |

**Table 2S**

| **a - Between-visits reproducibility** | | | | | | | | | | | | | | |
| --- | --- | --- | --- | --- | --- | --- | --- | --- | --- | --- | --- | --- | --- | --- |
|  | **NDI** | | | | | **ODI** | | | | | **FISO** | | | |
|  | **SB** | | **MB2** | **MB3** | | **SB** | | **MB2** | **MB3** | | **SB** | | **MB2** | **MB3** |
| **BCC** | 0.95 | | 0.91 | 0.85 | | 0.87 | | 0.95 | 0.96 | | 0.58 | | 0.83 | 0.88 |
| **GCC** | 0.81 | | 0.85 | 0.77 | | 0.93 | | 0.95 | 0.96 | | 0.86 | | 0.87 | 0.84 |
| **CST** | 0.89 | | 0.75 | 0.64 | | 0.99 | | 0.99 | 0.98 | | 0.36 | | 0.59 | 0.40 |
| **EC** | 0.93 | | 0.90 | 0.90 | | 0.83 | | 0.94 | 0.97 | | 0.29 | | 0.86 | 0.31 |
| **OR** | 0.95 | | 0.97 | 0.94 | | 0.75 | | 0.97 | 0.97 | | 0.28 | | 0.23 | 0.63 |
| **FL** | 0.80 | | 0.85 | 0.76 | | 0.89 | | 0.87 | 0.68 | | 0.96 | | 0.96 | 0.95 |
| **OL** | 0.87 | | 0.88 | 0.88 | | 0.92 | | 0.95 | 0.94 | | 0.70 | | 0.94 | 0.83 |
| **Caudate** | 0.81 | | 0.53 | 0.63 | | 0.56 | | 0.89 | 0.84 | | 0.90 | | 0.88 | 0.88 |
| **Putamen** | 0.90 | | 0.84 | 0.90 | | 0.87 | | 0.77 | 0.90 | | 0.21 | | 0.56 | 0.84 |
| **Thalamus** | 0.82 | | 0.64 | 0.55 | | 0.97 | | 0.96 | 0.95 | | 0.85 | | 0.95 | 0.81 |
| **Cerebellum** | 0.72 | | 0.76 | 0.74 | | 0.71 | | 0.85 | 0.91 | | 0.86 | | 0.94 | 0.95 |
| **b - Between-methods agreement** | | | | | | | | | | | | | | |
|  | **NDI** | | | | | **ODI** | | | | | **FISO** | | | |
|  | **SB-MB2** | **MB2-MB3** | | | **SB-MB3** | **SB-MB2** | **MB2-MB3** | | | **SB-MB3** | **SB-MB2** | **MB2-MB3** | | **SB-MB3** |
| **BCC** | 0.16 | 0.60 | | | 0.10 | 0.53 | 0.96 | | | 0.48 | 0.52 | 0.54 | | 0.24 |
| **GCC** | 0.15 | 0.73 | | | 0.11 | 0.47 | 0.90 | | | 0.32 | 0.70 | 0.92 | | 0.57 |
| **CST** | 0.07 | 0.60 | | | 0.05 | 0.49 | 0.95 | | | 0.40 | 0.00 | 0.16 | | -0.31 |
| **EC** | 0.12 | 0.56 | | | 0.08 | 0.38 | 0.86 | | | 0.24 | 0.09 | 0.62 | | -0.02 |
| **OR** | 0.27 | 0.86 | | | 0.19 | 0.31 | 0.83 | | | 0.21 | 0.01 | 0.35 | | 0.01 |
| **FL** | 0.41 | 0.66 | | | 0.22 | 0.18 | 0.38 | | | 0.09 | 0.94 | 0.84 | | 0.70 |
| **OL** | 0.31 | 0.93 | | | 0.25 | 0.34 | 0.69 | | | 0.18 | 0.25 | 0.87 | | 0.13 |
| **Caudate** | 0.13 | 0.42 | | | 0.08 | 0.26 | 0.73 | | | 0.22 | 0.85 | 0.80 | | 0.62 |
| **Putamen** | 0.14 | 0.47 | | | 0.08 | 0.28 | 0.72 | | | 0.15 | -0.02 | 0.66 | | -0.02 |
| **Thalamus** | 0.07 | 0.25 | | | 0.04 | 0.20 | 0.72 | | | 0.12 | 0.40 | 0.94 | | 0.47 |
| **Cerebellum** | 0.39 | 0.74 | | | 0.22 | 0.30 | 0.91 | | | 0.23 | 0.43 | 0.85 | | 0.31 |
