# Supplementary figures and images for: Comparing multiband and singleband EPI in NODDI at 3 T: what are the implications for reproducibility and study sample sizes?
Source: MAGMA. 2020 Dec 14;34(4):499–511. doi: 10.1007/s10334-020-00897-7 (PMC8338814; doi:10.1007/s10334-020-00897-7)

## Slide 1
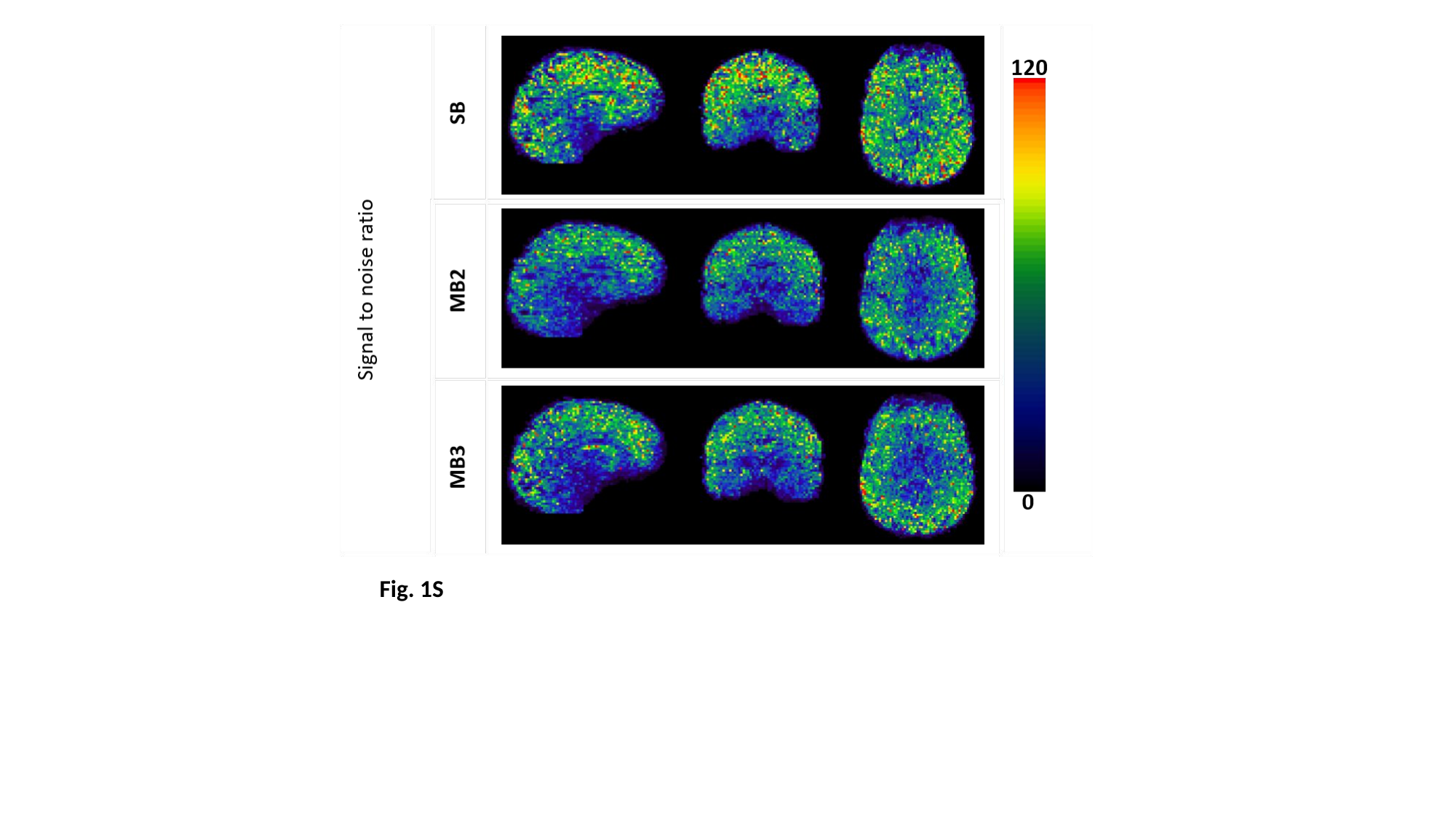

Fig. 1S

## Slide 2
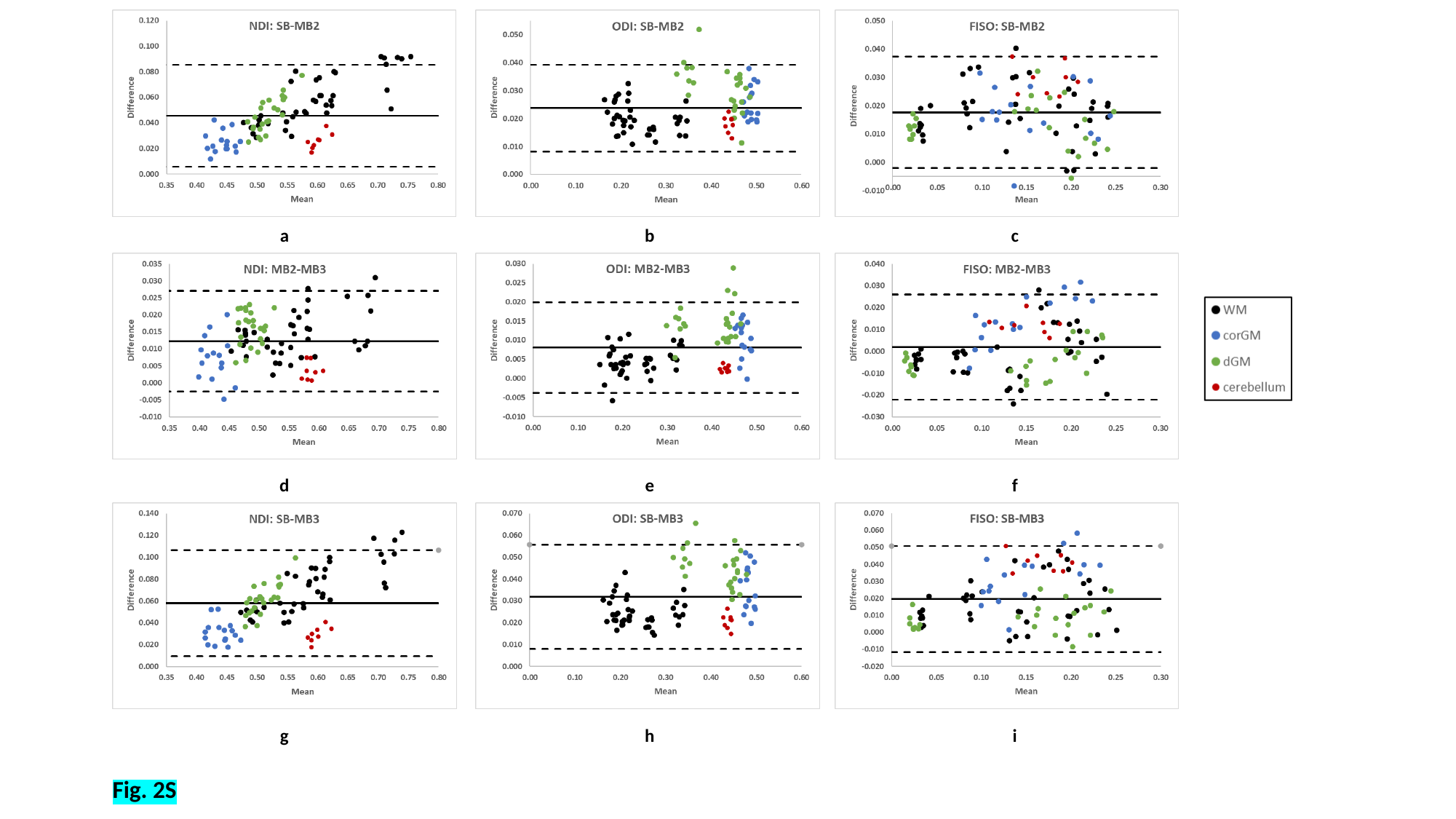

| | | |
| --- | --- | --- |
| a | b | c |
| | | |
| d | e | f |
| | | |
| g | h | i |
Fig. 2S

Supplement: Supplementary file 2 — Supplementary file2 Fig. 1S: Signal to noise ratio in diffusion signal for data acquired using Singleband EPI (SB) sequence, Multiband EPI factor 2 (MB2) and Multiband EPI factor 3 (MB3) (data taken from subject one during visit one). Fig. 2S: Bland-Altman plot of SB vs MB2 (a-c),MB2 vs MB3 (d-f), and SB vs MB3 (g-i) to estimate ODI, FISO and NDI measures (from visit 2) in all subjects for the following ROIs: Body of Corpus Callosum BCC, Genu of Corpus Callosum GCC, Corticospinal Tracts CST, External Capsules EC, Optic Radiation OR, Frontal Lobe FL, Occipital Lobe OL, Caudate, Putamen Thalamus and Cerebellum. SB = Singleband, MB2 = Multiband factor 2 and MB3 = Multiband factor 3. (Datapoints’ colour represents different tissues: black for white matter (WM), blue for cortical gray matter (corGM), green for deep gray matter (dGM) and red for the cerebellum). (PPTX 888 KB) [file 10334_2020_897_MOESM2_ESM.pptx]
